# Genomic signatures of inbreeding in a critically endangered parrot, the kākāpō

**DOI:** 10.1093/g3journal/jkab307

**Published:** 2021-08-31

**Authors:** Yasmin Foster, Ludovic Dutoit, Stefanie Grosser, Nicolas Dussex, Brodie J Foster, Ken G Dodds, Rudiger Brauning, Tracey Van Stijn, Fiona Robertson, John C McEwan, Jeanne M E Jacobs, Bruce C Robertson

**Affiliations:** 1 Department of Zoology, University of Otago, Dunedin 9054, New Zealand; 2 Centre for Palaeogenetics, SE-106 91 Stockholm, Sweden; 3 Department of Bioinformatics and Genetics, Swedish Museum of Natural History, SE-104 05 Stockholm, Sweden; 4 Department of Zoology, Stockholm University, SE-106 91 Stockholm, Sweden; 5 AgResearch Invermay Agricultural Centre, Mosgiel 9053, New Zealand; 6 AgResearch Lincoln Research Centre, Christchurch 8140, New Zealand

**Keywords:** conservation, genetic management, offspring survival, inbreeding coefficient, inbreeding depression, heterozygosity, runs of homozygosity

## Abstract

Events of inbreeding are inevitable in critically endangered species. Reduced population sizes and unique life-history traits can increase the severity of inbreeding, leading to declines in fitness and increased risk of extinction. Here, we investigate levels of inbreeding in a critically endangered flightless parrot, the kākāpō (*Strigops habroptilus*), wherein a highly inbred island population and one individual from the mainland of New Zealand founded the entire extant population. Genotyping-by-sequencing (GBS), and a genotype calling approach using a chromosome-level genome assembly, identified a filtered set of 12,241 single-nucleotide polymorphisms (SNPs) among 161 kākāpō, which together encompass the total genetic potential of the extant population. Multiple molecular-based estimates of inbreeding were compared, including genome-wide estimates of heterozygosity (F_H_), the diagonal elements of a genomic-relatedness matrix (F_GRM_), and runs of homozygosity (RoH, F_RoH_). In addition, we compared levels of inbreeding in chicks from a recent breeding season to examine if inbreeding is associated with offspring survival. The density of SNPs generated with GBS was sufficient to identify chromosomes that were largely homozygous with RoH distributed in similar patterns to other inbred species. Measures of inbreeding were largely correlated and differed significantly between descendants of the two founding populations. However, neither inbreeding nor ancestry was found to be associated with reduced survivorship in chicks, owing to unexpected mortality in chicks exhibiting low levels of inbreeding. Our study highlights important considerations for estimating inbreeding in critically endangered species, such as the impacts of small population sizes and admixture between diverse lineages.

## Introduction

Extensive inbreeding between close relatives and subsequent fitness effects are a major threat to the resilience of critically endangered populations (Charlesworth and Willis 2009; Frankham *et al.* 2017). Inbreeding is conventionally measured from pedigrees, but advances in DNA sequencing technologies have made it possible to study the consequences of complex intergenerational inbreeding at the scale of the genome across entire species or populations (Kardos *et al.* 2015a; Benazzo *et al.* 2017). Reduced-representation genome-wide sequencing allows for the cost-effective acquisition of single-nucleotide polymorphism (SNP) datasets that are well-suited for computationally efficient population genetic analyses (Baird *et al.* 2008; Andrews *et al.* 2016). Furthermore, best practices for population genetic analyses using SNPs continue to be refined with findings from simulations (Keller *et al.* 2011; Kardos *et al.* 2015a; Wang 2016) and empirical studies (Huisman *et al.* 2016; Grossen *et al.* 2018; McLennan *et al.* 2019). It is now feasible for conservation programs to routinely integrate genomic sequencing into management strategies (Shafer *et al.* 2015; Hendricks *et al.* 2018). Knowledge of population structure, relatedness, and levels of inbreeding can inform breeding decisions and provide crucial insight into the future viability of endangered populations (Allendorf *et al.* 2010). It is therefore important to evaluate the performance of different marker-based measures of inbreeding and understand how they relate to fitness-associated traits such as offspring survival (Keller 1998; Fu *et al.* 2019).

Reduced fitness in offspring of related parents is known as inbreeding depression and results from increases in homozygosity in two genetically distinct ways. These include the increase and exposure of homozygous recessive alleles maintained at low frequencies by mutation-selection balance, and the increase of homozygous alleles at loci exhibiting heterozygous advantage (*i.e.*, overdominance) maintained at moderate frequencies by balancing selection (Charlesworth and Willis 2009; Frankham *et al.* 2017). Inbreeding and resulting increases in homozygosity (*i.e.*, exposure of harmful mutations) can elevate extinction risk in endangered populations through increased susceptibility to disease (Benton *et al.* 2018; Townsend *et al.* 2018), reduced population growth rates (Bozzuto *et al.* 2019), higher prevalence of congenital defects (Ralls *et al.* 2000; Robinson *et al.* 2019), and reduced reproductive success (Keller 1998). In small populations, there is also concern that inbreeding, as well as demographic and environmental stochasticity, can act in combination with genetic drift to limit adaptive potential (Kimura 1957; Hoffmann *et al.* 2017; Díez-del-Molino *et al.* 2018; Leroy *et al.* 2018; Mable 2019). However, it has also been suggested that in populations that experience reduced effective population sizes over long periods of time, some deleterious alleles can be purged through a combination of inbreeding and purifying selection (Hedrick and Garcia-Dorado 2016; Caballero *et al.* 2017).

Recent empirical and simulation studies demonstrate that inbreeding estimates from genomic approaches are more precise and less downwardly biased compared to traditional pedigree-based methods (Keller *et al.* 2011; Taylor *et al.* 2015; Kardos *et al.* 2015a; Wang 2016). Pedigree-based estimates of inbreeding predict the expected proportion of an individual’s genome that is identical-by-descent (IBD), but low variances and the inability to measure stochastic effects such as linkage and Mendelian segregation can hamper the ability to detect inbreeding effects (Keller *et al.* 2011; Knief *et al.* 2017; Kardos *et al.* 2018). In addition, large multigenerational pedigrees are difficult to obtain for wild populations and may be impractical for long-lived species or species with urgent conservation needs (Kardos *et al.* 2016). Numerous studies have demonstrated the utility of inbreeding estimates generated from microsatellites, targeted-gene sequencing, reduced-representation sequencing, and whole-genome sequencing (Hoffman *et al.* 2014; Knief *et al.* 2015; Huisman *et al.* 2016; Norén *et al.* 2016; Humble *et al.* 2018; Lemopoulos *et al.* 2019; McLennan *et al.* 2019). Other studies have evaluated the number and depth of genome-wide markers necessary to apply equitable population genetic analyses (Kardos *et al.* 2015a, 2018), and have compared the effects of bioinformatic pipelines on population genetic inferences; in particular genotype callers and filtering options (Andrews *et al.* 2016; Benestan *et al.* 2016; Paris *et al.* 2017; Shafer *et al.* 2017; O’Leary *et al.* 2018; Díaz-Arce and Rodríguez-Ezpeleta 2019). With these recent advances, and the increasing availability of genome-wide SNP data, genomic methods are ripe for integration into the conservation management of endangered populations (Wright *et al.* 2020).

The critically endangered kākāpō (*Strigops habroptilus*) is endemic to New Zealand, and unique among parrots in that it is flightless, nocturnal, and possesses a polygynous lek mating system (Powlesland *et al.* 2006). Kākāpō underwent a significant population decline following the introduction of mammalian predators and other anthropogenic impacts, with a single male (Richard-Henry) surviving from the mainland of New Zealand (Powlesland *et al.* 2006; Dussex *et al.* 2018). Kākāpō were rediscovered on Stewart Island (∼30 km south of New Zealand’s South Island) and a small founding population of 61 individuals were translocated (of which 39 have reproduced), together with the single remaining mainland male, to predator-free offshore islands from the late 1970s onwards (Powlesland *et al.* 1995). The impacts of originating from a small insular population, having an extended life span with infrequent breeding, and possessing a lek mating system where a dominant male can father most of the offspring, predisposes the kākāpō to inbreeding (Clout and Merton 1998; Robertson 2006; Merton *et al.* 1984). For instance, one male kākāpō (Blades) from the Stewart Island founding population has fathered 22 chicks (of which 18 survived) since being translocated to predator-free islands, representing a significant genetic contribution to the total population size of 201 adults (as of August 2021). Indeed, previous studies exploring the recent evolutionary history of kākāpō found a ∼30-fold decline in genetic diversity within the mitochondrial genomes of historical and modern individuals (Bergner *et al.* 2016; Dussex *et al.* 2018). Reduced genetic diversity and inbreeding depression within kākāpō manifests as a consequential number of early-death embryos, smaller clutch sizes, and reduced hatching success, consistent with a low number of effective breeders (Bergner *et al.* 2014; White *et al.* 2015). Current management strategies to mitigate inbreeding include preventing consanguineous matings and promoting matings involving kākāpō descended from the mainland founder (Robertson 2006; Bergner *et al.* 2014).

Estimating inbreeding from pedigree-based methods in kākāpō would be futile, as their pedigrees are confounded by unknown parentage in the founding population and deep intergenerational consanguineous matings (Robertson 2006). Furthermore, pedigree analysis assumes that founders of the population are unrelated, which is unlikely to be true for the small founding island population of kākāpō. In this study, we use a reduced-representation genotyping-by-sequencing (GBS) approach (Elshire *et al.* 2011; Dodds *et al.* 2015) to revise measures of inbreeding in kākāpō, which were previously estimated from microsatellites (Bergner *et al.* 2014; White *et al.* 2015). Nearly all adult kākāpō since the translocation of the founding population in the late 1970s, up to and including chicks from the 2016 breeding season were included in genotyping. Together, these individuals encompass the total genetic potential of the extant kākāpō population. Deceased chicks and adults, and a single early-death embryo, were also genotyped. Discovery of SNPs was facilitated by mapping of GBS reads to a high-quality genome assembly of a kākāpō (Jane) provided by the Vertebrate Genome Project (Dussex *et al.* 2021; Rhie *et al.* 2021), allowing a rigorous reference-based approach (Shafer *et al.* 2017; O’Leary *et al.* 2018). We calculated multiple estimators of inbreeding in kākāpō, including genome-wide estimates of heterozygosity (F_H_) and the diagonal elements of a genomic-relatedness matrix (GRM, F_GRM_). Several studies using whole-genome sequencing have demonstrated that runs of homozygosity provide the most robust estimator of genome-wide patterns of inbreeding (*i.e.*, autozygosity) (Kardos *et al.* 2017, 2018). Therefore, we also screened for runs of homozygosity (RoH, F_RoH_) in the GBS dataset and evaluated its accuracy relative to F_H_ and F_GRM_. In addition, levels of inbreeding were compared between the descendants of the mainland and Stewart Island founding populations to further investigate the impacts of previous bottlenecks (Dussex *et al.* 2018). Finally, we compared inbreeding estimates between deceased and surviving kākāpō chicks from a recent breeding season to understand potential genetic factors underlying premature mortality (Fu *et al.* 2019).

## Materials and methods

### Study population and management

The total extant population of kākāpō is intensively managed by the kākāpō Recovery Team of the New Zealand Department of Conservation. Kākāpō were thought to be functionally extinct prior to the 1970s (Clout and Merton 1998). Between 1974 and 1977, 18 surviving males were discovered in Fiordland on the mainland of New Zealand, but only a single male (Richard-Henry) survived and contributed to the current managed population (Clout and Merton 1998; Powlesland *et al.* 2006); Richard-Henry and his descendants are referred to as the mainland founder and descendants hereafter (*n *=* *10). In 1977, a small insular population on Stewart Island was also rediscovered and eventually translocated (*n *=* *61) to predator-free islands (Lloyd and Powlesland 1994; Powlesland *et al.* 1995); this translocated population and its descendants are referred to as Stewart Island founders and descendants hereafter (*n *=* *153). Some relationships between individuals of the founding population are currently unresolved (Robertson 2006), and recent genomic data indicate that the Stewart Island population constitutes a distinct lineage that has been separated from the mainland population for thousands of generations since the last ice age (Dussex *et al.* 2021). The current population (as of August 2021) totals 201 individuals maintained on New Zealand predator-free islands: Whenua Hou (Codfish Island), Te Hauturu-o-Toi (Little Barrier Island), Te Kākahu-o-Tamatea (Chalky Island), and Anchor Island. The majority of kākāpō have been repeatedly transferred between these islands as part of management, and therefore the current location of individuals was not considered in our analyses. Capture, handling, and sample collection were performed in accordance with ethical requirements approved by Ngāi Tahu and the New Zealand Department of Conservation.

### DNA sequencing

Kākāpō samples were obtained as blood and stored in lysis buffer (Seutin *et al.* 1991) until DNA extraction, or as tissue and stored in absolute ethanol at −20°C. Adult kākāpō were sampled exclusively from blood (*n *=* *138), and a selection of blood, chorioallantoic membrane, and liver were sampled from surviving and deceased chicks from the 2016 breeding season (*n *=* *38). Genomic DNA was isolated from blood or tissue using standard phenol-chloroform extractions following Sambrook *et al.* (1989). GBS was performed at AgResearch Invermay, New Zealand, closely following Elshire *et al.* (2011) and Dodds *et al.* (2015). Kākāpō GBS libraries were double-digested with restriction enzymes PstI and MspI (NEB R140L and R0106L, New England Biolabs, Ipswich, USA), with even digestion and no evidence of repetitive elements in Bioanalyser traces (2100 Bioanalyser, Agilent Technologies, Santa Clara, USA). Following ligation of barcoded adapters, libraries were pooled and multiplexed. Amplification was followed by purification and size selection performed on a Pippin (193–500 bp, SAGE Science, Beverly, USA; 2% agarose, dye-free with internal standards CDF2050, Marker L CDF2010). Each GBS library, consisting of 94 samples, was run on a single lane of an Illumina HiSeq2500 generating single-ended reads for 101 cycles in high-output mode (v4 chemistry).

### SNP calling and filtering

Stacks v1.46 *process_radtags* was used to demultiplex raw reads and trim barcodes (Catchen *et al.* 2013; Paris *et al.* 2017; Rochette and Catchen 2017). Trimming of additional Illumina adaptors and removal of low-quality bases (Q < 20) were performed using *trim_galore* v0.4.5, a tool combining *Cutadapt and Fastqc* (Martin 2011; Andrews *et al.* 2012; Krueger 2015). All reads were truncated to the same length of 72 bp with *Cutadapt* v2.3 while optimizing the number of reads written (Martin 2011). Finally, *MultiQc* v1.5 was used to collate the quality control information after every read processing step before mapping to the reference genome and SNP calling (Ewels *et al.* 2016). *Burrow Wheelers Aligner* v0.7.15 (algorithm *BWA mem*; Li 2013) was used with default parameters for the alignment of preprocessed GBS reads to the kākāpō reference genome. Briefly, the chromosomal-level assembly identified 24 autosomes and *ZW* sex-chromosomes; total length 1165.62 Mb, N50 scaffold 83 Mb, and N50 contig 9.5 Mb (NCBI: GCA_004027225.1) (Dussex *et al.* 2021; Rhie *et al.* 2021). The assembly allowed for a rigorous reference-based approach where reads were mapped to a chromosome-level assembly before SNP calling, resulting in decreased type I errors (Davey *et al.* 2011; Shafer *et al.* 2017). After alignment, *Samtools* v1.8 (*view*, *sort*, *and* *flagstat*) was used to convert and sort SAM files to BAM files, and to print statistics for checking alignment mapping rate (Li *et al.* 2009).

*Stacks* v1.46 was used to call SNPs with a reference genome (Catchen *et al.* 2013; Paris *et al.* 2017; Rochette and Catchen 2017). Default parameters were used for *ref_map.pl* except to include a reduction in soft clipping during *pstacks* (–max_clipped 0.5). *Stacks populations* with default parameters and filtering for one SNP per RAD locus (–write_single_snp) was used to output genotypes in VCF and PLINK formats for further downstream analyses (Catchen *et al.* 2013). After SNP calling, a total of 14 individuals with a high proportion of missing data (>70%) were removed, as well as one female Stewart Island founder (Jean), following obscure parentage results in previous microsatellite datasets (B. Robertson, unpublished data), which were reproduced in initial population structure analyses in the current study (results not shown). Variants were then filtered per individual in *VCFtools* v1.14 (Danecek *et al.* 2011) for a minimum read depth of two and a maximum read depth of 30 (–minDP 2, –maxDP 30) across genotypes to reduce repetitive elements and allowing up to 20% missing data (–max-missing 0.80), as to not remove excessive numbers of markers while taking into consideration founder ancestry and the limited diversity within the kākāpō genome (Huang and Lacey Knowles 2016; Shafer *et al.* 2017; O’Leary *et al.* 2018; Ahrens *et al.* 2021). The *Z*-chromosome and *W*-chromosome were removed from the dataset using *VCFtools*, so that only autosomal markers were used for downstream analyses. Genetic diversity can be biased when filtering for high minor allele frequencies (MAF), since these markers can overestimate the proportion of heterozygous sites (Ekblom *et al.* 2018), and strong MAF filtering increases the downward bias of inbreeding and relatedness estimates (Weir and Goudet 2017; Goudet *et al.* 2018). Therefore, we did not filter for MAF explicitly during SNP calling; some downstream softwares, however, can incorporate MAF filtering (*e.g.*, *KGD*; Dodds *et al.* 2015). Scripts for methods described here are available from https://github.com/yasfoster/kakapo_gbs.

A principal component analysis (PCA) based on filtered SNPs was conducted in *PLINK* v1.9 and *R* (Purcell *et al.* 2007; R Core Team 2020) to rule out the presence of unexpected population structure. Two highly divergent founding populations, previously identified by Dussex *et al.* (2018, 2021), were apparent in the PCA (Supplementary Figure S1). Consequently, three variations of the data were filtered separately after excluding different subsets of individuals, to consider potential effects of population substructure on inbreeding estimates. Briefly, a main dataset containing all individuals was filtered for minimum and maximum depth, and for missing data, leaving a total of 12,241 SNPs for 161 individuals. Identical filtering was applied to a second dataset after the removal of the sole mainland founder Richard-Henry and his only three offspring (F1) prior to estimating inbreeding, leaving a total of 12,089 SNPs for 157 individuals. Finally, identical filtering was performed on a third dataset after the removal of all mainland descendants, Richard-Henry and both his F1 and F2 descendants (*n *=* *10), leaving 12,207 SNPs for 151 individuals.

### Measures of inbreeding

Estimating individual inbreeding is strongly influenced by the number of SNPs called, the variance explained by markers, and the expected heterozygosity within the population (Kardos *et al.* 2015a; Knief *et al.* 2017). To meet theoretical requirements for detecting inbreeding depression, markers should have nonzero variance and heterozygosity should correlate with the heterozygosity of functional loci; this phenomenon is termed identity disequilibrium (ID) (Weir and Cockerham 1973; Szulkin *et al.* 2010). We characterized the extent of variation in inbreeding and the degree to which markers reflect genome-wide heterozygosity using the *inbreedR* v0.3.2 package in *R*, with bootstrapping (*n *=* *1000; Stoffel *et al.* 2016). ID was quantified using *g2*, a metric that reflects how heterozygosity is correlated across markers, whereby significant mean *g2* values provide support for variance in inbreeding in the population (Szulkin *et al.* 2010; Stoffel *et al.* 2016). We also calculated heterozygosity–heterozygosity correlation coefficients to estimate ID by dividing the SNP markers into two random subsets and computing the correlation in heterozygosity between them, with subsetting replicated (*n *=* *1000) (Balloux *et al.* 2004; Stoffel *et al.* 2016). The input variant files were formatted for *inbreedR* using the packages *vcfR* v1.10.0 and *reshape2* v1.4.4 in *R* (Wickham 2007; Knaus and Grünwald 2017).

The inbreeding coefficient (F_H_) (also known as F_HOM_, F_IS_, or F_PLINK_) and diagonal elements of the genomic relatedness matrix (F_GRM_) are relative measures of inbreeding within the total population, indicating the probability that an individual carries alleles that are identical by descent (IBD) (Wright 1969; VanRaden 2008). F_GRM_ (also known as $\hat{F}$^III^, F_UNI_, or F_ALT_) quantifies allelic similarity between gametes and gives more weight to homozygous rare alleles (Nietlisbach *et al.* 2019). Runs of homozygosity (F_RoH_) is an absolute measure of individual autozygosity: the realized proportion of the genome that is IBD (McQuillan *et al.* 2008). Inferences of population history can be made based on the distribution of RoH length; long RoH are indicative of recent inbreeding and arise from recent ancestry, whereas short RoH can result from background relatedness or indicate distant common ancestors (Kirin *et al.* 2010; Kardos *et al.* 2016; Ceballos *et al.* 2018). Short RoH are commonly disregarded when comparing samples within a modern population, as it is difficult to know whether they result from a previous bottleneck or from background relatedness; thus a cutoff of >1 Mb was used when identifying RoH (Pemberton *et al.* 2012).

#### Inbreeding coefficient, F_H_:

The inbreeding coefficient (F_H_), the probability that an individual carries two IBD copies of an allele at a given neutral locus, was calculated using *VCFtools* (–het) and confirmed with *PLINK* (–het) using method of moments (Purcell *et al.* 2007; Danecek *et al.* 2011). F_H_ is defined as,

$$F_{H}=\frac{\mathrm{ObsHom}-\mathrm{ExpHom}}{\backslash \#\mathrm{SNPs}-\mathrm{ExpHom}}$$

where *ObsHom* is the observed number of homozygous loci in an individual, *ExpHom* is the expected number of homozygous loci under Hardy-Weinberg equilibrium, and *#SNPs* is the number of markers called. F_H_ ranges from −1 to 1 and measures the excess number of observed homozygous genotypes relative to the mean expected homozygosity, and can be considered as a measure of inbreeding under nonrandom mating within a population (Keller and Waller 2002; Keller *et al.* 2011; Kardos *et al.* 2016). In a random mating population, F_H_ should be centered near zero, whereas positive F_H_ values indicate individuals whose parents are more closely related than expected with a deficiency of heterozygotes, and negative values imply the opposite (Wang 2014; Waples and Allendorf 2015).

#### Genomic-relatedness matrix, F_GRM_:

The genomic relatedness matrix (GRM) and its diagonal elements of self-relatedness (F_GRM_) uses allele frequencies to provide unbiased estimates of individual inbreeding while accounting for read depth in the genotype calls, implemented in the *R* package “*kinship using GBS with depth* *adjustment”* (*KGD* v0.9.5, Dodds *et al.* 2015). This fully corrected method of estimation (G_5_) described by Dodds *et al.* (2015) uses methods equivalent to VanRaden (2008), except that missing genotypes are not imputed. Genotypes are used for estimating inbreeding only if both alleles of a SNP may be scored (*i.e.*, if there are at least 2 reads). Individuals more inbred than average have positive values, whereas less inbred individuals are expected to have negative values. The Python script *vcf2ra.py* (available from https://github.com/AgResearch/KGD) was used to convert VCF to the “Tassel” format for input into *KGD*. Applying *KGD* filtering to the total dataset, 876 SNPs with a depth <0.01 or minor allele frequency (MAF) of zero were removed, leaving a total of 11,365 SNPs to construct the GRM; F_GRM_ = diagonal of GRM − 1. To identify regions containing repetitive elements, *KGD* provides a framework for further diagnostics by outputting plots illustrating SNP call rates and depth, MAF, and Hardy-Weinberg disequilibrium plotted against MAF for validation. This is referred to as a fin plot, which illustrates SNP average depth using a color gradient. For kākāpō, the fin plot showed that SNPs were concentrated in appropriate regions (Supplementary Figure S2A), with an intermediate depth across MAFs, and no large concentration of SNPs at the upper and lower boundaries; the latter suggesting no excess levels of heterozygosity. To investigate the influence of Hardy-Weinberg disequilibrium filtering on self-relatedness estimates, SNPs with deviations less than −0.05 were removed, which had a negligible effect on self-relatedness estimates (results not shown). *F_GRM_* is defined as,

$$F_{\mathrm{GRM}}=\sum_{j}\left(\frac{{\left(x_{j}-2p_{j}\right)}^{2}-8p_{j}(1-p_{j})K_{j}}{1-2K_{j}}\right)/2\sum_{j}p{}_{j}\left(1-p_{j}\right)-1$$

where *j* indexes SNPs with depth at least two in the individual, $K_{j}=1/2^{k_{j}}$, $k_{j}$ is the depth, ${x_{j}}_{\;}$ is the (inferred) number of reference alleles in the genotype, and $p_{j^{\;}}$ is the reference allele frequency (Dodds *et al.* 2015).

#### Runs of homozygosity, F_RoH_:

The accurate detection of runs of homozygosity (RoH) using reduced-representation sequencing approaches is highly dependent on read depth, SNP density, and the distribution of SNPs across the genome. However, dense markers from GBS and the availability of a high-quality chromosome-level genome assembly provide the necessary framework to map and compare RoH coordinates across the genome (Kardos *et al.* 2017; Ceballos *et al.* 2018; Grossen *et al.* 2018; Zhang *et al.* 2019). RoH analyses using reduced-representation sequencing may not necessarily provide the complete picture of autozygosity across the genome, particularly in regions with short RoH and low abundance of SNPs, making boundaries of RoH difficult to identify. However, RoH estimates derived from reduced-representation sequencing may be considered plausible estimates of inbreeding if they are congruent with traditional estimators of heterozygosity (*e.g.*, F_H_ and F_GRM_) (Kardos *et al.* 2015a). RoH was identified using the *–homozyg* function in *PLINK* (Purcell *et al.* 2007; Howrigan *et al.* 2011), setting the parameters to appropriate values for kākāpō (SNP density: 84.03 kb/SNP) while closely following Grossen *et al.* (2018) and Kardos *et al.* (2015, Supplementary Table S1). To account for occasional mutations or sequencing error, a single heterozygous position was allowed in inferred RoH. The following parameters were used to define RoH while ensuring that the edges of RoH are delimited: a minimum of 25 contiguous homozygous SNPs (–homozyg-snp 25), minimum SNP density of one SNP every 130 Kb (–homozyg-density 130), a maximum distance between neighboring SNPs of 1 Mb (–homozyg-gap 1000), and a maximum of one heterozygous site (–homozyg-het 1). Allowing up to three heterozygous sites (–homozyg-het 3), as suggested by Ceballos *et al.* (2018), did not impact the number of RoH found. In addition, the sliding window required >25 SNPs (–homozyg-window-snp 25), was defined as homozygous if it had a maximum of 1 heterozygous site (–homozyg-window-het 1) and allowed no more than 5 missing site calls (–homozyg-window-missing 5).

To calculate individual autozygosity or inbreeding (F_RoH_), a minimum length threshold of >1 Mb was required to qualify a RoH as homozygous in order to exclude RoH resulting from background relatedness or with strong linkage disequilibrium, which typically can extend into shorter regions of up to 100 Kb (McQuillan *et al.* 2008; Purfield *et al.* 2012). F_RoH_ is defined as,

$$F_{\mathrm{RoH}}=\frac{\sum L_{\mathrm{RoH}}}{L_{\mathrm{Auto}}}$$

where *∑ L_RoH_* is the sum of the total length of all of an individual's RoH, and *L_Auto_* the autosomal genome length (1028.67 Mb in kākāpō) (McQuillan *et al.* 2008). To compare alternative thresholds, F_RoH_ was additionally defined for long RoH >10 Mb (F_RoH10_). The F_RoH_ and F_RoH10_ estimates were compared between mainland and Stewart Island founders and descendants, and between deceased (*n *=* *9) and surviving chicks (*n *=* *25) from the 2016 breeding season.

### Chick survivorship

The inbreeding estimate F_RoH_ was compared between deceased and surviving chicks from the 2016 breeding season. We note, however, that chicks were removed from this comparison if they were deceased from known nonbiological causes (*n *=* *4); *e.g.*, crushed eggs, chicks deceased after conflict or drowning after a storm. The developmental stage was not considered, as age at death could not be resolved for all samples; thus, all samples are referred to as chicks hereafter. Differences in inbreeding between deceased (*n *=* *9) and surviving (*n *=* *25) chicks from the same breeding season were compared with F_RoH_ using the *lm* linear regression function in *R* (R Core Team 2020). A generalized linear model (GLM) was performed using the *glm* function in R with a binomial distribution and logit link function, with fixed predictor variables of ancestry (mainland or Stewart Island) and inbreeding (F_RoH_), and chick survivorship (dead or alive) as the response variable (*e.g.*, survival ∼ ancestry + F_RoH_), to evaluate if a relationship between ancestry, F_RoH_ and chick survival exists.

### Statistical analyses and visualization

To accurately measure the effects of inbreeding with SNPs, statistical power depends on the variation in inbreeding in a given population, the depth and accuracy of the SNPs called, as well as sample and effect sizes (Keller *et al.* 2011). Methods used to estimate inbreeding in this study have considered these criteria during parameter selection, such as subsetting the data, depth adjustment for the GRM (Dodds *et al.* 2015), and using ID to confirm there was nonzero variation in heterozygosity measures (Weir and Cockerham 1973; Szulkin *et al.* 2010). All statistical analyses and plotting were performed in *R Studio* v1.3.959, using the following packages: *ggplot2* v3.3.2*, ggpubr* v0.4.0*, ggfortify* v0.4.10, and *inbreedR* (Stoffel *et al.* 2016; Tang *et al.* 2016; Wickham 2016; Kassambara 2020; R Core Team 2020). The inbreeding estimates F_H_, F_GRM_, and F_RoH_ were compared with Pearson’s correlations using the *corr.test* in R (Schielzeth 2010; Kardos *et al.* 2018) using the three datasets described above. Differences in inbreeding between mainland and Stewart Island founders and descendants were compared with F_RoH_ using the *lm* linear regression function in *R*, since linear regression is robust to violations of the normality assumption (Knief and Forstmeier 2018).

## Results

**Figure 1:**
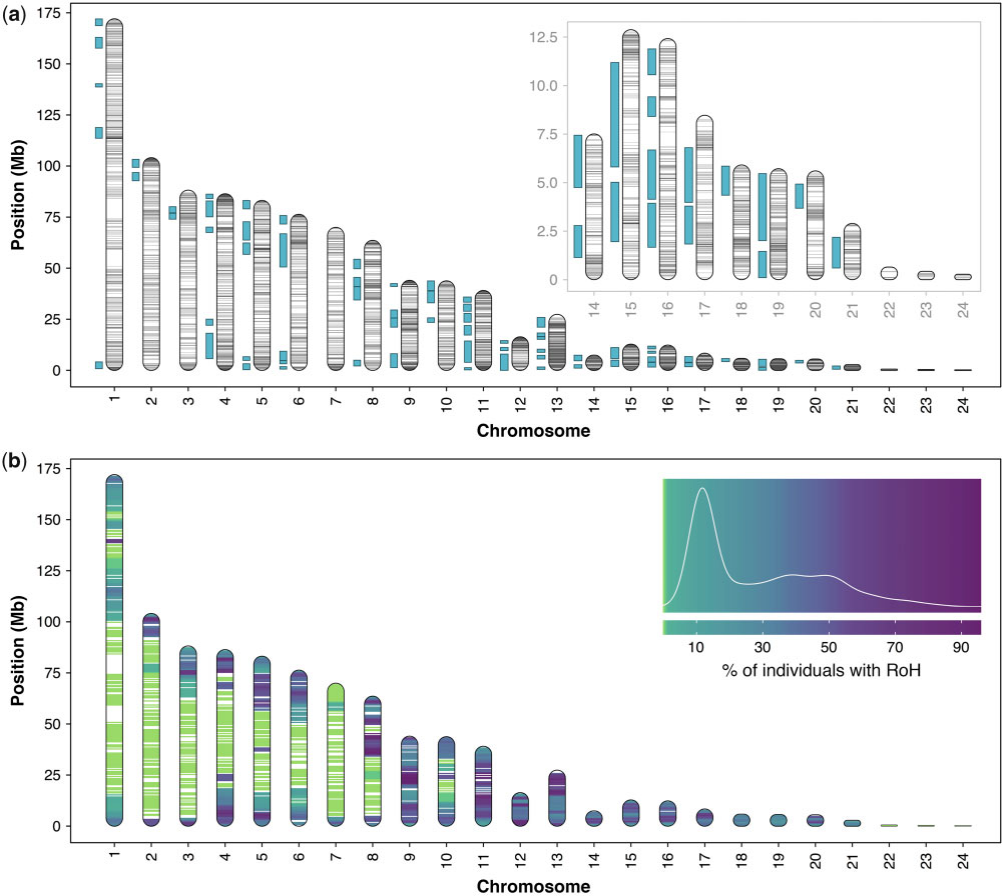
Autosomal chromosomes of the kākāpō reference genome illustrating distribution of single nucleotide polymorphisms (SNPs) and runs of homozygosity (RoH) after reduced-representation sequencing. (A) 12,241 SNPs are represented across chromosomes (gray horizontal lines), and RoH >1 Mb are represented adjacent to chromosomes (blue boxes) for an exemplary individual (Hillary) exhibiting excess homozygosity. Inset box displays a zoomed view of the last 11 micro-chromosomes. (B) RoH prevalence across the kākāpō genome among all individuals (*n* = 161) in nonoverlapping 500 Kb windows, with density plot representing a color gradient scaled to prevalence distribution of RoH.

We generated high-density genome-wide SNPs for 123 adult kākāpō and 38 chicks from the 2016 breeding season using GBS with reference-based genotype calling. Processed raw reads were aligned to a high-quality reference genome, resulting in a mean mapping rate of 98.51%. Calling SNPs with the referenced-based approach yielded 56,218 SNPs. After filtering for minimum and maximum depth, removing sex-linked markers and individuals with high SNP call missingness, and excluding variants with more than 20% missing data, 12,241 SNPs with a mean depth of 9.82 and density of 84.03 kb/SNP remained. After applying KGD filtering, a total of 11,782 SNPs with a mean depth of 9.55 were used to construct the GRM for all individuals (diagonal elements are shown in Supplementary Figure S2B). Autosomal SNPs were distributed across all chromosomes and concentrated toward the ends of the chromosomes (Figure 1A). Mean observed homozygosity of the markers across all Stewart Island descendants was 73.5%, and 62.8% for mainland descendants, and significantly differed between descendant groups (*P* < 2.26 × 10^−11^) (Supplementary Figure S3). The PCA revealed no unexpected population structure; the mainland founder and its descendants separated from the Stewart Island founders consistent with their ancestry (Supplementary Figure S1).

**Figure 2:**
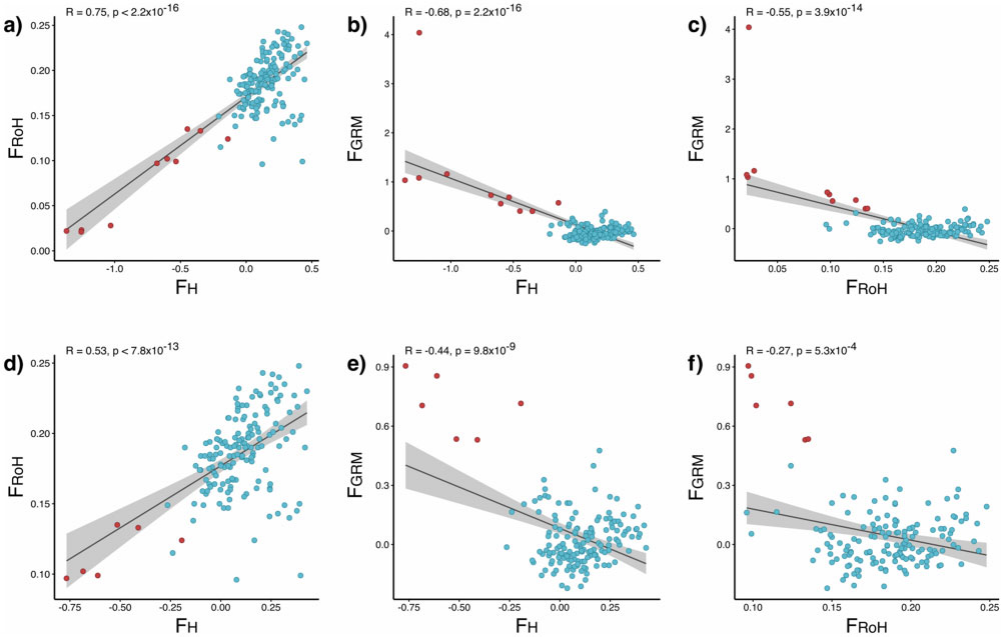
Correlations between inbreeding estimates for all kākāpō (*n* = 161): (A) F_H_ and F_RoH_, (B) F_H_ and F_GRM_, and (C) F_RoH_ and F_GRM_. Pearson’s *r- and P*-values are above each plot. Blue points represent Stewart Island-only descendants and red points represent mainland descendants. Correlations between inbreeding estimates for all kākāpō except for the sole mainland founder and his three only offspring (*n* = 157): (D) F_H_ and F_RoH_, (E) F_H_ and F_GRM_, and (F) F_RoH_ and F_GRM_.

We compared multiple estimates of genome-wide inbreeding among all kākāpō. First, ID using the metric *g2* confirmed nonzero variance (*g2 *=* *0.11 ± 0.02 [SE], *P* = 0.01; Supplementary Figure S4A), with a strong positive heterozygosity-heterozygosity correlation coefficient (*r* = 0.989; Supplementary Figure S4B), indicating that the SNP markers meet the requirements to detect inbreeding depression (Szulkin *et al.* 2010; Stoffel *et al.* 2016). Using the three subsets of data, we then compared the three inbreeding estimates: F_H_, F_RoH_, and F_GRM_. For the total dataset including all individuals, the inbreeding coefficient F_H_ was most strongly correlated with F_RoH_ (Pearson’s *r* = 0.75, *P* < 2.2 × 10^−16^; Figure 2A), and moderately but inversely correlated with F_GRM_ (Pearson’s *r* = −0.68, *P*  <  2.2 × 10^−16^; Figure 2B). F_RoH_ and F_GRM_ were also moderately but inversely correlated with each other (Pearson’s *r* = −0.55, *P* = 3.9 × 10^−14^; Figure 2C). For the dataset excluding the mainland founder and his three offspring, the inbreeding coefficient F_H_ remained most strongly correlated with F_RoH_ (Pearson’s *r* = 0.53, *P* < 7.8 × 10^−13^; Figure 2D), and moderately correlated with F_GRM_ (Pearson’s *r* = −0.44, *P* = 9.8 × 10^−9^; Figure 2E). However, F_RoH_ and F_GRM_ were only weakly correlated (Pearson’s *r* = −0.27, *P* = 5.3 × 10^−4^; Figure 2F). For the dataset containing no mainland founder or descendants, the inbreeding coefficient F_H_ was moderately correlated with F_RoH_ (Pearson’s *r* = 0.36, *P* < 7.7 × 10^−6^; Supplementary Figure S5A) but no correlations were found between F_H_ and F_GRM_ (Pearson’s *r* = 0.092, *P* = 0.26; Supplementary Figure S5B), or F_RoH_ and F_GRM_ (Pearson’s *r* = 0.09, *P* = 0.27; Supplementary Figure S5C). For all kākāpō, the mean F_H_ was 0.09, mean F_GRM_ was 0.04, and mean F_RoH_ was 0.18 (Supplementary Figure S6, A–C). When the Stewart Island and mainland descendants were considered separately (within the total dataset), the Stewart Island mean F_H_ was 0.15, mean F_GRM_ was −0.03, and mean F_RoH_ was 0.19 (Supplementary Figure S7, A–C). For the mainland descendants, mean F_H_ was −0.77, mean F_GRM_ was 1.07, and mean F_RoH_ was 0.08 (Supplementary Figure S7, A–C).

**Figure 3:**
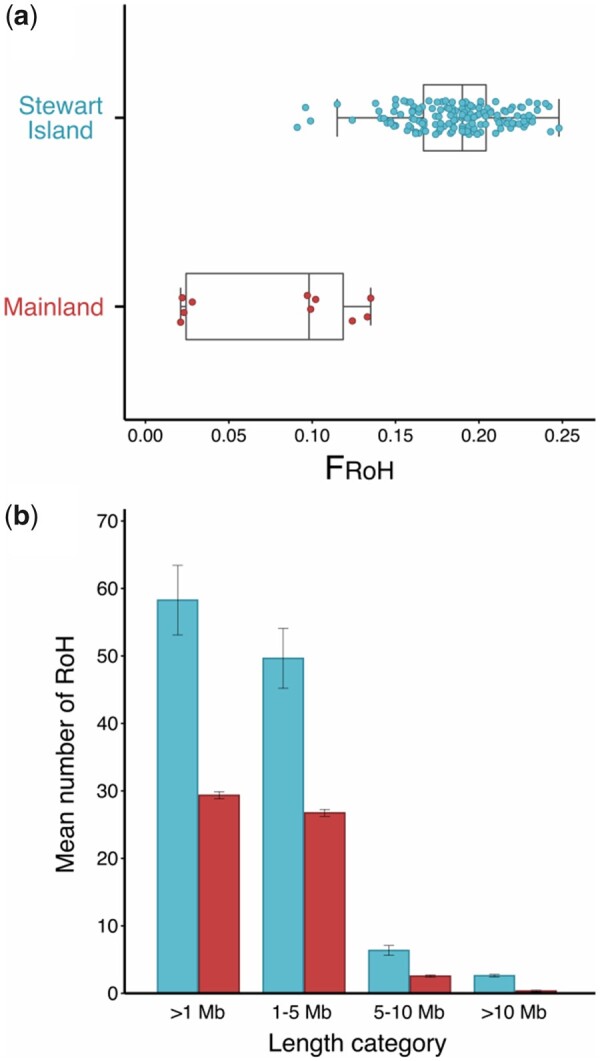
Distribution of individual autozygosity for all kākāpō using runs of homozygosity (RoH) >1 Mb (*n* = 161): (A) Boxplots comparing F_RoH_ between Stewart Island and mainland descendants. (B) Mean number of RoH for each length category. Error bars represent standard error. Blue points and bars represent Stewart Island-only descendants, and red points and bars represent mainland descendants.

Inbreeding was estimated among kākāpō descendant groups using RoH along the autosomal genome (Figure 1, A and B). RoH were found across the genome up to and including chromosome 21 (Figure 1B; Supplementary Figure S8A). No RoH were detected on the remaining microchromosomes, perhaps because they are too short for RoH >1 Mb to accumulate or because the recombination rate is too high (Figure 1, A and B). In most kākāpō, some chromosomes were almost completely covered with RoH (*e.g.*, chromosome 11, 15, and 19 for male kākāpō Hillary; Figure 1A). Scanning for RoH that were >1 Mb in length containing at least 25 SNPs found a total of 9,372 RoH across all individuals, among which shorter segments between 1 and 5 Mb predominated (Figure 3B). The mean number of RoH per individual was 56.49 and mean length of RoH was 185.55 Mb. When considered by descendant groups, the Stewart Island individuals had a mean number of 58.30 RoH and mean length of 192.50 Mb (16.46% of genome in RoH), and the mainland descendants had a mean number of 29.20 RoH and mean length of 80.63 Mb (6.92% of genome in RoH) (Supplementary Figure S8, B and C). We additionally divided RoH into size categories indicative of the probable timing of their formation (McQuillan *et al.* 2008; Kirin *et al.* 2010). Of 9,372 RoH >1 Mb, 7,995 RoH were between the length of 1−5 Mb (85% of RoH, ∼10−50 generations), 991 RoH between the length of 5−10 Mb (11% of RoH, ∼5−10 generations), and 385 RoH >10 Mb (4% of RoH, ∼5 generations) (Figure 3B) (Howrigan *et al.* 2011; Xu *et al.* 2019). For inbreeding estimated from RoH >1 Mb, the mean F_RoH_ for Stewart Island descendants was 0.19 and 0.08 for mainland descendants (Figure 3A; Supplementary Figure S7C). For inbreeding estimated from RoH >10 Mb, which are associated with recent inbreeding events, the mean F_RoH_10_ for Stewart Island was 0.03, and for the mainland descendants was 0.002 since all but one individual had no RoH >10 Mb (Supplementary Figure S7D). In addition, a linear regression showed a significant difference between the F_RoH_ of mainland and Stewart Island descendants (*P* < 2 × 10^−16^) (Figure 3A).

**Figure 4:**
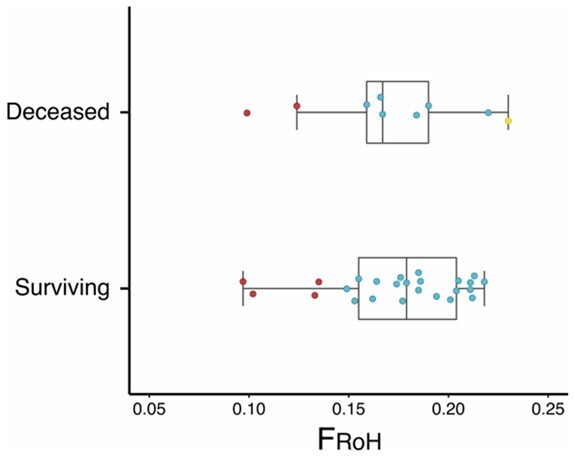
Distribution of individual autozygosity (F_RoH_) between surviving and deceased kākāpō chicks using runs of homozygosity (RoH) >1 Mb. Blue points represent Stewart Island-only chicks, red points represent mainland chicks, and the yellow point represents a chick with suspected dwarfism.

We used F_RoH_ to compare inbreeding between deceased (*n *=* *9) and surviving (*n *=* *25) chicks from the 2016 breeding season. Deceased chicks had a mean number of 55.33 RoH with a mean length of 175.75 Mb (15.08% of genome in RoH), and surviving chicks had a mean number of 55.92 RoH with a mean length of 180.26 Mb (15.46% of genome in RoH). The number of RoH did not significantly differ between deceased and surviving chicks (*P* = 0.87) or between length of RoH (*P* = 0.75). There was no significant relationship between chick survival and their F_RoH_ (*P* = 0.76) or F_RoH10_ (*P* = 0.46; Figure 4). In addition, a GLM indicated that there were no significant effects of ancestry or F_RoH_ on chick survivorship (Supplementary Table S1). Similar to the mainland founder (Richard-Henry) and his three offspring, all but one of his descending grand-chicks (F2) had no RoH >10 Mb. Notably, despite the absence of a difference in F_RoH_ between surviving and deceased chicks, the single mainland descendant chick that had two RoH >10 Mb was an early-death embryo. Furthermore, one chick suspected of dwarfism also had the highest value of F_RoH_ out of all deceased chicks (Figure 4). Comparing the two different ancestral groups, chicks descending from Stewart Island individuals had a mean number of 58.82 RoH with a mean length of 192.1 Mb, and chicks descending from mainland individuals (F2) had a reduced mean number of 41.50 RoH and shorter mean length of 118.22 Mb. The number of RoH (*P* = 6.25e-07) and length of RoH (*P* = 3.32e-08) per individual significantly differed between Stewart Island and mainland descendant chicks. F_RoH_ (*P* = 3.39e-08; Figure 4) and F_RoH10_ (*P* = 0.006) were both significantly different between Stewart Island and mainland descending chicks.

## Discussion

Reduced-representation sequencing across the genome (*e.g.*, GBS) is a cost-effective approach to evaluate inbreeding in populations under conservation management (Narum *et al.* 2013; Andrews *et al.* 2016). Here, we used multiple genome-wide estimates to examine patterns of inbreeding in the kākāpō, as pedigree-based methods are limited by depth (generations) and statistical power, and cannot accurately predict what proportion of the genome is IBD ( Kalinowski and Hedrick 1999; Keller *et al.* 2011; Forstmeier *et al.* 2012; Kardos *et al.* 2015a). Genome-wide inbreeding estimates are particularly advantageous for kākāpō since pedigree information is incomplete for the founders of the current population, whose relationship and age are unknown, and who descend from two distinct ancestral populations (Bergner *et al.* 2016; Dussex *et al.* 2018, 2021). A total of 12,241 high-quality filtered SNPs were found in the dataset, which contained the majority of adults (*n *=* *123) and chicks from the 2016 breeding season (*n *=* *38), representing virtually the total managed kākāpō population up until 2018.

SNPs were distributed across the kākāpō genome and were concentrated toward the ends of chromosomes (Figure 1A), consistent with known patterns of recombination within bird genomes (Backström *et al.* 2010; Ellegren 2010; Murray *et al.* 2017). Signatures of inbreeding were highly conspicuous across the genome, with entire micro-chromosomes almost completely covered with RoH in some individuals (*e.g.*, chromosomes 11, 15, and 19; Figure 1, A and B), reflecting extreme levels of homozygosity previously found in kākāpō (Dussex *et al.* 2021). Comparable levels of homozygosity are found in a highly inbred Scandanavian gray wolf population in which entire chromosomes are completely autozygous (Kardos *et al.* 2018). In *Ficedula* flycatchers, humans, and livestock, RoH are more abundant in regions of the genome with low nucleotide diversity and recombination, and in regions subject to strong purifying selection (Pemberton *et al.* 2012; Curik *et al.* 2014; Kardos *et al.* 2017; Ceballos *et al.* 2018). High rates of recombination break up haplotype blocks to generate increasingly shorter tracts of homozygosity, whereby shorter RoH are indicative of background relatedness or inbreeding arising from distant common ancestry, and long RoH are signatures of recent parental relatedness or occur in regions with low rates of recombination (McQuillan *et al.* 2008; Pemberton *et al.* 2012). The majority (85%) of RoH in kākāpō ranged between 1 and 5 Mb (Figure 3B), suggesting that the excess homozygosity observed in the modern population originates from both inbreeding experienced by distant common ancestors and resulting background relatedness of recent generations (Pemberton *et al.* 2012; Kardos *et al.* 2015a). The distribution of RoH on chromosomes of kākāpō is consistent with the concentrated distribution of SNPs toward chromosome ends (Figure 1B), where shorter RoH are known to occur in regions of high recombination (Pemberton *et al.* 2012). Estimates of F_RoH_ based on long RoH (*i.e.*, >10 Mb) may be more powerful for detecting inbreeding depression (Kardos *et al.* 2015a), but their ascertainment from reduced-representation sequencing may be impacted by insufficient numbers of SNPs within certain genomic regions (*i.e.*, by long RoH being broken up into short RoH). A limited number of long RoH were found in kākāpō using this approach (Figure 3B). The complete genomic architecture of inbreeding in kākāpō may be further resolved with comparisons using whole-genomes and corresponding mutational load, as well as identifying RoH deserts and islands (*i.e.*, hotspots) (Pemberton *et al.* 2012; Curik *et al.* 2014). Indeed, using historical kākāpō genomes, Dussex *et al.* (2021) found an 8.5-fold increase in F_RoH_ (>2 Mb) in a subset of the extant Stewart Island population compared to the extinct mainland population (including Richard-Henry).

Estimates of individual inbreeding levels remained correlated across the datasets containing all kākāpō (Figure 2, A–C) and with the mainland founder and his three offspring excluded (Figure 2, D–F). However, when all individuals with mainland ancestry were excluded, only F_H_ and F_RoH_ remained correlated with each other (Supplementary Figure S5A), revealing that the majority of variation in inbreeding levels in kākāpō is driven by differences between the two divergent founding populations. The weakest correlations between inbreeding estimates invariably involved GRM methods (F_GRM_), which use estimated population allele frequencies and are highly influenced by what initial population is provided (*e.g.*, Figure 2, C and F). This method may not be appropriate for kākāpō considering the extreme subdivision between mainland and Stewart Island founders, with additional ascertainment bias due to having only one mainland founder. Indeed, previous studies have found that inbreeding estimates from RoH are more accurate for smaller populations (low N_e_), as GRM-based approaches give too much weight to rare alleles causing biases when there are subdivided populations and admixture between individuals with diverse allele frequencies (Nietlisbach *et al.* 2019; Alemu *et al.* 2021; Caballero *et al.* 2021). In kākāpō, weighting of rare alleles results in greater F_GRM_ values for mainland descendants because of their homozygosity for rare alleles, whereas Stewart Island descendants are homozygous for common alleles (VanRaden 2008).

The inbreeding coefficient F_H_ was significantly correlated with F_RoH_ (Figure 2, A and D; Supplementary Figure S5A), suggesting that high-quality GBS datasets contain sufficient signal to estimate genomic IBD in the absence of whole-genome data (Allendorf *et al.* 2010). Kardos *et al.* (2018) found that F_RoH_ measured from the whole genomes of gray wolves were strongly correlated with F_RoH_ estimated from as few as 10,000 randomly subsampled SNPs across the genome (*r*^2^ = 0.97); comparable recommendations are given by Allendorf *et al.* (2010: 10,000 SNPs) and Gervais *et al.* (2019: 7,000 SNPs). Negative F_H_ values result from excess heterozygosity relative to Hardy-Weinberg proportions and indicate that parents are, on average, less closely related than expected under random mating (Keller and Waller 2002; Kardos *et al.* 2016, Box 2). F_H_ values in kākāpō were more negative for the mainland founder (Richard-Henry) and its descendants than for Stewart Island descendants (Supplementary Figure S7A), indicating that individuals with mainland ancestry are relatively more outbred within the extant population. Both F_H_ and F_RoH_ similarly reflected elevated levels of inbreeding in Stewart Island descendants (Supplementary Figure S7, A and C), and overall, F_RoH_ estimates were elevated (maximum F_RoH_ 0.248) compared to those estimated from RADseq data of the vulnerable New Zealand hihi (maximum F_RoH_ 0.158) (Duntsch *et al.* 2021). Mainland and Stewart Island descendants differed significantly in their values of F_RoH_, with mainland descendants possessing shorter and fewer RoH. Mainland descendants also had a lower number of RoH longer than 10 Mb, indicating that less significant inbreeding had occurred recently in the ancestral population (Figure 3, A and B). Studies examining founder-specific inbreeding depression suggest that the magnitude of eventual inbreeding depression is influenced significantly by initial relatedness levels in the population, amounts of introgression, and variation among founders that exists due to the segregation of large-effect deleterious recessive alleles (Lacy *et al.* 1996; Allendorf *et al.* 2010). For example, heightened inbreeding depression in the Hawaiian crow (‘Alalā) was found to originate from a single pair that initially founded the captive breeding population (Hedrick *et al.* 2016). Signatures of inbreeding in kākāpō suggest that founder-specific effects are ongoing, with inbreeding estimates in descendants of two distinct ancestral populations remaining consistent across multiple generations. Furthermore, founder-specific effects are likely to increase in magnitude due to the extended lifespan, long generation time, and lek mating system of kākāpō, where certain individuals from the founding population continue to contribute disproportionately to matings.

Genetic rescue aims to increase fitness in endangered populations through the introduction of unrelated individuals, with demonstrated success in numerous species (Whiteley *et al.* 2015; Bell *et al.* 2019). Under this paradigm, we expected that offspring with lower levels of inbreeding would exhibit signs of increased fitness as a result of heterosis or hybrid vigor (Charlesworth and Willis 2009). Specifically, it was expected that mainland-descending chicks, which have mixed ancestry and lower levels of inbreeding, would exhibit greater survivorship compared to chicks descending from Stewart Island ancestry only. Inbreeding was strongly associated with ancestry but did not have an effect on chick survival (Figure 4), with neither inbreeding (F_RoH_) nor ancestry predicting survivorship (Supplementary Table S1). This pattern was driven by mortality in chicks descended from mainland ancestry, despite mainland descendants exhibiting the lowest levels of inbreeding in the population, as well as mortality in Stewart Island chicks exhibiting both high and moderate levels of inbreeding. Dussex *et al.* (2021) recently found that mainland kākāpō had a higher mutational load than individuals from Stewart Island, suggesting that deleterious alleles may have been removed from the Stewart Island population through a combination of genetic drift and purging. Limited evidence for inbreeding depression in our study may potentially also be explained by the dynamics of purging and alleviation of some of the effects of inbreeding. We note, however, that detection of inbreeding depression using comparisons of offspring survivorship (and other fitness traits) in critically endangered species are often limited by statistical power and sample size. Further evidence should be obtained using kākāpō chicks from subsequent breeding seasons.

Current management strategies to mitigate inbreeding in kākāpō include the prevention of consanguineous matings, removal of infertile or overly successful males from breeding islands, and favoring matings with mainland descendants (Elliott *et al.* 2001; Robertson 2006; Bergner *et al.* 2014). Full-sibling and parental-offspring matings already naturally occur due to disproportionately successful males and the lek mating system of kākāpō (Eason *et al.* 2006; Bergner *et al.* 2014). For instance, one male kākāpō (Blades) from the Stewart Island founding population has fathered 22 chicks (of which 18 survived), and between 1991 and 1999 another male founder (Felix) fathered 7 of a total 13 chicks (Miller *et al.* 2003; Eason *et al.* 2006). Current strategies that favor matings between mainland and Stewart Island descendants could have unforeseen consequences for population viability. For example, the introduction of a single immigrant male to the Isle Royale wolf population initially appeared advantageous but ultimately did not mitigate intensive inbreeding depression and now its imminent extinction, highlighting how deleterious mutations hidden in a large outbred population can be detrimental once introduced to a smaller inbred population (Hedrick *et al.*^2014^, ^2019^).

Although genetic rescue is an appropriate strategy for inbred species when alternate populations are available for acquiring genetic diversity (Ralls *et al.* 2020), source populations carrying a low risk of causing outbreeding depression no longer exist in many endangered species (Kyriazis *et al.* 2021). In kākāpō, the consequences of introducing potentially harmful mutations from the single mainland descendant (Richard-Henry) into the recovering extant population (Dussex *et al.* 2021), which has remained consistently small enough for purging to take place (Robinson *et al.* 2019; Hoffmann *et al.* 2021), potentially challenges the benefits of genetic rescue within kākāpō conservation management. Specifically, increases in homozygosity could have exposed deleterious large-effect alleles to selection, thereby removing them from the Stewart Island population and reducing the impact of inbreeding on fitness (Hedrick 1994; Wang *et al.* 1999; Keller and Waller 2002); although weakly deleterious alleles might still impact individual fitness (*i.e.*, genetic load) (Grossen *et al.* 2020; Mathur and DeWoody 2021). A study on the Chatham Island black robin, for example, revealed improved fledging success for chicks from highly inbred mothers (Weiser *et al.* 2016), suggesting that purging of some of the mutational load may have occurred. We recommend that ongoing conservation management in kākāpō should focus on detecting individuals exhibiting inbreeding depression and monitoring the effects of mainland ancestry on the population (*e.g.*, F3 hybrids between mainland and Stewart Island). Outcomes of strategies such as translocations and artificial insemination may be improved if a greater emphasis is placed on the selection of individuals that carry desirable alleles for breeding (to minimize deleterious variation) rather than individual relatedness alone (Kardos and Shafer 2018).

Markers of homozygosity can be used to detect causal mutations associated with malformations and disease (Kardos *et al.* 2016). In the California condor, the mutation underlying chondrodystrophy, a lethal form of dwarfism, is yet to be identified. However, traditional pedigree analysis indicates that an autosomal recessive allele is likely to be responsible (Ralls *et al.* 2000). In kākāpō from the 2016 breeding season, an individual with signs of chondrodystrophy possessed the highest F_RoH_ value of all deceased chicks (Figure 4). Mapping approaches based on RoH offer new avenues to discover loci contributing to inbreeding depression and recessive monogenic diseases (Kijas 2013; Ceballos *et al.* 2018). High-density SNP markers capable of reliably defining RoH may yield new candidate loci for malformations in inbred populations, such as chondrodystrophy in the Californian condor and kākāpō, and vertebral defects in Isle Royale wolves (Robinson *et al.* 2019). Deleterious alleles in genes associated with immunity may also be subject to purging (*e.g.*, toll-like receptors, Nelson-Flower *et al.* 2018), and should be considered in subsequent homozygosity mapping in kākāpō. Future investigations into diseases affecting kākāpō (*e.g.*, cloacitis, aspergillosis) should also incorporate homozygosity mapping and targeted-gene approaches to identify susceptible individuals and minimize their exposure to sources of transmission.

GBS provided congruent estimates of inbreeding across the kākāpō genome based on relative (F_H_) and absolute measures of autozygosity (F_RoH_). Future studies should compare estimates of inbreeding with additional quantitative phenotypic traits (*e.g.*, clutch size and birth weight) to further evaluate evidence for inbreeding depression in kākāpō (Hoffman *et al.* 2014; Bérénos *et al.* 2016; Huisman *et al.* 2016). Inbreeding estimates can also be incorporated into a number of other methods, including analysis of: linkage disequilibrium (Bersabé *et al.* 2015; Humble *et al.* 2018), haplotype inference (Leitwein *et al.* 2020), selective sweeps (Kardos *et al.* 2015b, 2017; Qanbari *et al.* 2019), homozygous deleterious genotype enrichment (Szpiech *et al.* 2019), inbreeding-related patterns of DNA methylation (von Holdt *et al.* 2017), and nucleotide diversity (π) for estimating adaptive potential (Dutoit *et al.* 2017; de Villemereuil *et al.* 2019; Mable 2019). As whole-genome resequencing data becomes available for kākāpō, the dynamics between inbreeding depression (White *et al.* 2015) and the reduction of deleterious alleles through purging or drift (Dussex *et al.* 2021) should be further evaluated. Furthermore, resulting inferences should be compared with the present study to assess the computational- and cost- burden of whole-genome sequencing (Kardos and Shafer 2018). Measures of homozygosity and autozygosity offer critical insight into the consequences of inbreeding in endangered populations, with important implications for conservation management.

## Data availability

Genome assembly available from BioProject ID: PRJNA489135, NCBI assembly: GCA_004027225.1. Genotype data (VCF files) and corresponding results are available on figshare at https://doi.org/10.6084/m9.figshare.15113106. Scripts are available from https://github.com/yasfoster/kakapo_gbs and https://github.com/AgResearch/KGD. Supplementary material is available on figshare: https://doi.org/10.25387/g3.14626371.
